# Nursing interventions to the person with cardiac disease submitted to ECMO – integrative literature review

**DOI:** 10.1080/07853890.2021.1896014

**Published:** 2021-09-28

**Authors:** João Reisinho, João Sardinha, Pedro Santos, Rita Rodrigues, Cidália Castro

**Affiliations:** a Nurse Department, Escola Superior de Saúde Egas Moniz (ESSEM), Egas Moniz Cooperativa de Ensino Superior, Caparica, Portugal; b Centro de Investigação Interdisciplinar Egas Moniz (CiiEM), Egas Moniz Cooperativa de Ensino Superior, Caparica, Portugal; c Unidade de Investigacão e Desenvolvimento em Enfermagem (UI&DE), Lisboa, Portugal

**Keywords:** Cardiac disease, critical patient, ECMO, nursing interventions

## Abstract

**Introduction:**

Heart disease includes a variety of diseases, conditions, and disorders that affect the heart and blood vessels [1]. Extracorporeal Membrane Oxygenation (ECMO) is a short-term Ventricular Assist Device (VAD), indicated in cardiogenic shock and respiratory failure, when surgical or conventional therapeutic measures are not successful, and the most used in the case of heart failure is venoarterial ECMO [2,3]. The aim of this study is to understand the ECMO technique in adult patients with cardiac disease, as well as the associated Nursing Interventions.

**Materials and methods:**

This study is an Integrative Literature Review by the PI[C]OD method. Available articles were searched in the Biblioteca do Conhecimento Online (B-On) and EBSCO*host* databases, published between 2014 and 2019. The inclusion criteria were ECMO, person with cardiac disease and successfully nursing interventions. Of the total of twenty-seven articles, 14 were excluded by title, six by the abstract and four by full reading. Three articles were included in the corpus of the study.

**Results:**

The articles included allowed to identify three main ideas: in the first article, a specific nursing care protocol was developed for patients with VAD [4]; in the second study, VADs can be an intermediary mean for cardiac transplantation [5]; however, in the last study, ECMO may present physical complications and depressive symptoms [2].

**Discussion and conclusions:**

Studies have shown that in patients with unstable cardiac pathology “ECMO is life saving, and should therefore be considered a viable extension for conventional treatment options for critical care” [3]. Although VAD is a reliable alternative to transplantation, when it is not available or when the patient's condition is hemodynamically unstable, heart transplantation remains the first-line treatment [5]. We think that the results of this study can subsidise the exercise of teaching nursing care, allowing students to build structured knowledge, which allows to identify the real needs of the person submitted to ECMO. At the level of clinical practice, it is important to discuss the results of this study within the team, ensuring patient safety and the quality of nursing care based on scientific evidence.
